# Evaluation of a Problem-Solving Program on Developmental Disorders: A Qualitative Appraisal

**DOI:** 10.7759/cureus.51679

**Published:** 2024-01-04

**Authors:** Nanami Murata, Soma Sagara, Rinhi Higashiguchi, Yukine Hori, Yi Ning Tan, Yasuhiro Kotera, Akihiko Ozaki

**Affiliations:** 1 Postgraduate Clinical Training Center, Wakayama Medical University Hospital, Wakayama, JPN; 2 Faculty of Medicine, Osaka University, Suita, JPN; 3 Graduate School of Education, The University of Tokyo, Tokyo, JPN; 4 School of Medicine, Tokyo Women’s Medical University, Tokyo, JPN; 5 Faculty of Medicine & Health Sciences, University of Nottingham, Nottingham, GBR; 6 Surgery, Medical Governance Research Institute, Tokyo, JPN; 7 Breast Surgery, Jyoban Hospital of Tokiwa Foundation, Iwaki, JPN

**Keywords:** stigma, inclusiveness, student, education, project-based learning, developmental disorders

## Abstract

Background

In Japan, effective educational methods to promote understanding of developmental disorders (DD) among young people have not been established, something that is necessary for an inclusive society.

Aims

This paper aimed to identify both the positive aspects and areas for improvement in a problem-solving program for middle and high school students on the topic of DD called inochi Gakusei Innovators Program (i-GIP). The study also sought to determine the changes in attitudes toward DD that occurred as a result of participating in the program.

Method

Semi-structured interviews were conducted online, with middle and high school students who participated in i-GIP, university students who helped manage the program, and cooperators with DD or their families. Inductive thematic analysis was conducted, and codes and themes were identified.

Results

Positive aspects of i-GIP included its project-based learning approach, raising awareness and understanding of developmental disorders, and the proactive attitude of the students. Areas for improvement in the program were identified, including program administration and addressing challenges related specifically to DD. Changes in attitudes and behavior toward DD were reported, along with improvements in interpersonal relationships.

Conclusions and implications

This study suggests that incorporating a project-based approach can be a useful manner to learn about DD among young people.

## Introduction

As many as 15% of the world's population lives with some form of disability [1], and in order to enable diverse people to enjoy a high quality of life (QOL), it is essential that all citizens gain an understanding of disability, as well as how to interact with persons with disabilities. The United Nations adopted the “Convention on the Rights of Persons with Disabilities” in 2006 [2] and in Japan, the “Act for the Elimination of Discrimination against Persons with Disabilities” was enacted in 2016 and revised in 2021 [3]. It became a legal obligation for the national government, local governments, and private businesses to provide reasonable accommodation for persons with disabilities. In reality, however, a lack of understanding of disability, as well as disparities caused by disability still exist. In particular, people with developmental disorders (DD) have been reported to feel more isolated and less socially connected [4], and to have a lower QOL [5,6].

DD are behavioral and cognitive disorders that arise during the developmental period that involve significant difficulties in the acquisition and execution of specific intellectual, motor, language, or social functions [7]. It is estimated that globally, there are 52 million children under the age of five with DD [8]. DD are not only difficult to identify, but even once identified, the lack of easily observable DD traits makes it difficult to promote understanding of persons with DD among the people around them [9]. For school-aged children, one of the DD-related issues is the high rate of truancy. While the general rate of truancy is 0.32% in elementary school and 2.89% in middle school, the percentage of patients diagnosed with DD who are found to be truant is 44.3% [10]. Here, interpersonal problems with friends and teachers are often cited as triggers for non-attendance. Another pertinent issue is the high turnover rate during employment. Despite the fact that the employment rate of persons with disabilities has been increasing yearly [11], the turnover rate within one year for persons with DD is 37.5%, compared to the 19.4% turnover rate for new graduates in the general population [12,13]. Again, interpersonal problems are cited as the reason for the high turnover rate [13]. The understanding of DD is not yet widespread in Japan.

In order to deepen societal understanding of DD, it is essential to educate the middle/high school student population [14]. These students not only have sufficient cognitive ability but are also highly interested as well as greatly influential in society. It has been reported that traditional education regarding DD is often limited to promoting the self-understanding of the individuals concerned, and the understanding of their families and supporters; it is difficult to educate students without disabilities because these disabilities are “hard to spot” [15]. In other words, there is no established means of promoting understanding of DD among middle/high school students in Japanese educational settings.

Therefore, we, the inochi WAKAZO Project (hereafter referred to as inochi), conducted a problem-solving program on DD in 2020 (inochi Gakusei Innovators Program 2020, hereafter referred to as i-GIP). This study aims to identify positive aspects and areas for improvement in i-GIP and to clarify what changes in attitudes toward DD have occurred as a result of i-GIP. The study consists of interviews with the middle/high school students who participated in i-GIP, the university students who managed the program, as well as the persons with DD (PWDD) and their families who cooperated with us. The goal of this study is to evaluate this program as a way to promote the understanding of disability. In this paper, we looked into three key questions to comprehensively examine the impact of i-GIP. Firstly, we sought to identify the positive aspects of i-GIP. Secondly, we focused on identifying areas of improvement. Finally, we investigated changes in attitudes toward DD that have occurred in participants and cooperators as a result of i-GIP.

## Materials and methods

Overview of the project

What is the inochi WAKAZO Project?

The inochi WAKAZO Project is a student organization whose mission is “to create a society that saves lives through the power of youths”. There are approximately 130 university student members throughout Japan. Most of the members are medical students, but there are also students from other faculties.

**Figure 1:**
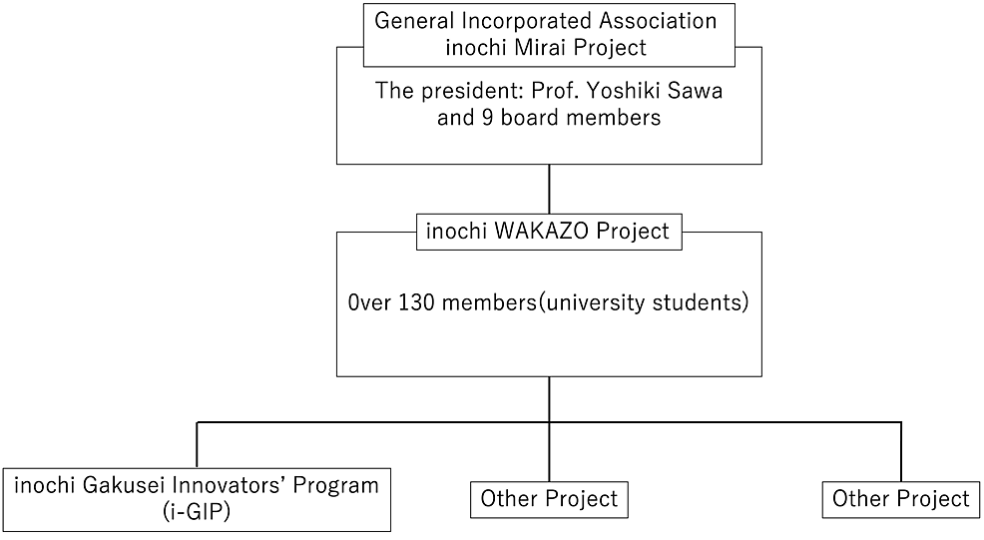
Organization chart of the inochi Mirai Project

The parent organization of the inochi WAKAZO Project is the inochi Mirai Project, a generally incorporated association consisting of 10 directors, some of whom are university professors (Figure 1). The project itself is managed entirely by university students, but the board members provide consultation on administrative matters, such as fundraising and public relations.

What Is i-GIP2020？

One of the main activities of the inochi WAKAZO project is i-GIP (inochi-Gakusei Innovators Program). i-GIP is a problem-solving program offered to middle/high school students. The program began in 2015, making 2020 its sixth year. Each year, the university students who manage the program decide on a theme related to a critical healthcare issue, and participating students aim to come up with problem-solving ideas related to the theme. In response to the growing attention on DD in Japan in recent years, the theme for 2020 was "The co-creation of a society where no one with developmental disorders is left behind”.

How Did We Conduct i-GIP2020?

We recruited participants, mainly from elite schools across Japan, through distributing flyers, publicizing the program on social media, and holding information sessions. Participants applied in teams of two to four members. A total of 140 teams (434 students) sent in applications, and after an interview and selection process, 33 teams (115 students) were invited to participate in i-GIP. Fifty-five university students were involved in i-GIP's management. Details of the selection process are as follows: two university students worked as a pair and served as interviewers. The pair then conducted a 20-minute interview for each team using ZOOM (Zoom Video Communications, California, USA). Each interviewer gave a score out of 100 points based on items such as enthusiasm, the ability to think logically, and the ability to take the initiative. Taking these scores into consideration, the university students discussed which teams to select.

Through a four-month-long program, each team of participants identified issues related to DD and created and implemented solutions for their selected issues. One university student mentor was assigned to each team, accompanying the students in their activities. The overview of the program is shown in Table 1. A total of 10 lectures on DD and design thinking were conducted during the program. Each lecture took about six hours and was conducted online mainly. An objective was pre-established by the management, and corresponding guest lecturers were invited to present and/or aid individual groups. Firstly, 3/4 guest lecturers who were PWDD were invited to talk about their experiences and work in the community. The goal was for participants to be able to put themselves in the shoes of people with DD. To aid in their understanding of DD, a handbook about DD was given out to all participants in the first lecture. This handbook was created by management under the supervision of experts. Several lectures were dedicated to deciding on an issue for groups to tackle. Workshops by design-thinking professionals and guest lectures by real-life initiatives tackling issues of DD were conducted. This allowed participants to have a rough overview of the whole problem-solving process, and how issues of DD looked, and how solutions could be implemented. There were opportunities for PWDD and professionals working with DD to provide feedback on issues and solutions of individual groups. Lectures on prototyping and implementation were also provided. In addition to the lectures, i-GIP strongly encouraged participants to personally interview PWDD and experts in DD. The goal was for participants to be able to determine crucial DD issues, create meaningful solutions for PWDD, implement them, and receive feedback. At the end of the program, there was a presentation competition where each team presented their solutions.

**Table 1:** Overview of i-GIP2020 DD: Developmental Disorders, PWDD: Persons with Developmental Disorders

Month	Objectives	Content
July	Acquire knowledge of DD	Lecture on how to conduct interviews
	Be able to put oneself in PWDD’s shoes	Lecture by PWDD
August	Identify and select an issue to tackle	Workshops by design-thinking instructors
	Learn how to create prototypes	
September	Determine a solution	Introduction of case studies of solutions to social issues
October	Implement solutions	Presentation and competition
	Present results	

Method and participants

This study employed a qualitative design with thematic analysis of semi-structured interviews. The interviews were conducted using ZOOM approximately one year after the program ended. The interview participants comprised eight middle or high school students who participated in the program, seven university students from the management team, and six people either with DD or who were family members of PWDD, hereafter also referred to as cooperators. Inclusion criteria were people who had participated or were involved in i-GIP. Individuals excluded from the study comprised the four authors of this paper who also served on the management team. A message was sent using pre-existing contacts (e.g. emails) explaining our study and that we were recruiting participants. Those who were interested then replied via email, upon which we sent them explanatory documents and consent forms. Written consent was obtained from all participants and participants under 20 years of age had written consent from their parents or guardians. Subsequently, we conducted interviews with those who demonstrated understanding of and agreement with the research content.

All questions were asked in a single interview, meaning the questions were not spread across multiple interviews, and each interview lasted for 30-50 minutes. After 21 interviews, the researchers confirmed that data saturation was achieved. Four of the authors of this article conducted the interviews, and all participants were aware that the interviewers were involved in the management of i-GIP. The interviewers conducted the interviews from their homes in Japan and confirmed that no one else was around to ensure confidentiality. All interview data were audio-recorded.

Interview guide

Aside from the basic information of the participants (age, gender, and affiliation), the interview guide focused on three main questions: 1) positive aspects of i-GIP, 2) areas of improvement of i-GIP, and 3) changes in the understanding of DD, both before and after i-GIP, as well as one year after the end of i-GIP. Four of the authors created this interview guide.

Data analysis

Inductive thematic analysis [16] was chosen due to its exploratory nature, allowing us to explore this relatively unexamined area (i.e. our research question and target population group in Japan): no themes were identified in advance. The social constructionism approach was undertaken to establish shared understandings of i-GIP with the participants. The analysis was divided among the four authors (NM, SS, RH, and YH) and each followed the six phases recommended by Braun and Clarke. 

First, we transcribed the data and requested all the participants to check the summarized contents, following COREQ (COnsolidated criteria for REporting Qualitative research). Then, we read and re-read the data to get ourselves familiarized with the data and noted down initial ideas that summarized the interviews (Phase 1). Next, we systematically identified features of the data across the entire data set and collated data relevant to each code (Phase 2). Following that, we gathered similar codes and created a higher level of themes (Phase 3). All authors checked the themes in relation to the coded extracts and the entire data set (Phase 4). After several discussions to clarify the definitions and names of each theme (Phase 5), we finalized the analysis as reported in this paper (Phase 6). The results of our analysis were again reviewed and agreed upon by all researchers.

Analysis was performed manually. No software was used. Finally, the transcripts and the final output of our analysis were shown to all participants, who confirmed the accuracy of the results.

## Results

The reasons for participation in i-GIP, the positive aspects of i-GIP, the areas for improvement in i-GIP, and the changes in attitudes and behavior toward DD before and after participation in i-GIP are described in this order, corresponding to the research questions. A total of 21 people were interviewed: eight middle/high school student participants, seven university students from the management team, and six cooperators (Supplementary Material 1).

Reasons for participation

Reasons for participation extracted from each group are shown in Table 2.

**Table 2:** Reasons for participation in three groups

Three Sample Groups	Category
Middle/High School Students	The program was intriguing
	Had time because of COVID-19
	Interest in the topic
	Influenced by an acquaintance/ Participation of acquaintances
University Students	The program was intriguing
	Had time because of COVID-19
	Interest in the topic
	Influenced by an acquaintance/ Participation of acquaintances
Cooperators	The program was intriguing
	High hopes for students/youth

In terms of reasons for participation, there were four reasons: “The program was intriguing”, “Had time because of COVID-19”, “Interest in the topic”, and “Influenced by an acquaintance/Participation of acquaintances”. These were common reasons for middle/high school participants and university students. On the other hand, among the cooperators, some motives were similar to middle/high school and university students, such as “The program was intriguing”, while others were not seen in the other two groups, such as “High hopes for students/youth”.

Table 3 summarizes the positive aspects of i-GIP, areas for improvement in i-GIP, as well as changes in attitudes and behavior toward DD before and after participation in i-GIP, along with the themes and comments for each group. Themes were numbered with the following rules: (Qa-b-Tc) where Qa (a= 1, 2, 3) corresponds to question number, b corresponds to an attribute of interviewee (H: Middle/ High school students, U: University students, C: Cooperators) and c (c= 1, 2, …) refers to number c of themes extracted from this question and attribute.

**Table 3:** Summary of the results DD: Developmental Disorders, PWDD: Persons with Developmental Disorders, H: Middle/High School Students, U: University Students, C: Cooperators, Q: Question, T: Theme

RQ	Three Groups	Theme	Comments
Good points	Middle/High School students	Characteristics of program (Q1-H-T1)	I was glad I got to listen to many lectures not only on DD but also on how to make presentations and prototypes and how to realize them. (H5)
		Understanding of developmental disorders (Q1-H-T2)	Through speaking to parents of children with DD, I realised many things about these children's experiences. The ones facing these difficulties are the children. When their parents/adults with DD asked if I had attempted to see things from the children's perspectives, I realised that that was something I had to do. I realised the importance of trying to put myself in the shoes of the relevant parties. (H6)
		Personal growth (Q1-H-T3)	There were many good things about working with people from other schools. It was more interesting when the groups were mixed. For interviews, not only could we widen the scope of our investigation, but we could also interview a variety of people from schools with different environments/cultures. This helped us to stay open-minded. (H3)
	University Students	Characteristics of program (Q1-U-T1)	The easiest part (of society) to bring about change is middle and high school students, yet what DD specialists find most difficult when trying to raise awareness is "how to target schools". Until now, approaching teachers has been the most they could do. GIP targets middle and high school students. (U1)
		Understanding of developmental disorders (Q1-U-T2)	Both middle/high school students and university students conducted interviews. I had an image of DD as reported in media, but after actually talking to them, I realized that it was different from what is portrayed in media. Hearing about first-hand experiences was truly eye-opening for me. (U5)
		Personal growth (Q1-U-T3)	I become more accepting of diversity. (U4)
	Cooperators	Use of project-based learning (Q1-C-T1)	What I found most wonderful was that the students did not just listen passively but were proactive and engaged (in communicating with PWDD). (C1)
		Raising awareness and understanding (Q1-C-T2)	As a parent, I am reassured to know that the students who are trying to tackle and are paying attention (to DD) will be the future leaders of the next generation. (C6)
		Attitude of students (Q1-C-T3)	It was great to learn that there are people who really care about DD. (C1)
Improvements	Middle/High School students	Challenges related to developmental disorders (Q2-H-T1)	Some PWDD do not want to meet strangers or cannot accommodate sudden requests. I think conducting interviews was more difficult in comparison to previous years. (H2)
		Administration (Q2-H-T2)	Although we had no choice because of the pandemic, it would have been nice to be able to do it (GIP) in person. (H8)
		Capacity of participants (Q2-H-T3)	The only area for improvement I can think of is a personal issue. I thought it was unfortunate that I only participated in my second year of high school then stopped (when deciding whether to continue working on the project after GIP) because of exams. (H4)
	University Students	Challenges related to developmental disorders (Q2-U-T1)	We could only interview people who face difficulties in daily life, who have self-understanding, and can talk about their experiences to others. (U4)
		Administration (Q2-U-T2)	In terms of education for understanding disabilities, it is questionable whether it was good to rank the participants' proposed solutions. It is also true that competition is required for motivation. They were ranked according to whether it was feasible as a business, but the rankings do not reflect the severity of the problem. (U2)
		Capacity of participants (Q2-U-T3)	That I had to sacrifice sleep to complete tasks. (U4)
	Cooperators	Administration (Q2-C-T1)	I don't know what they did with it (the projects) after that, or what happened after the award was given. (C6)
		Improvements solution-wise (Q2-C-T2)	I also felt that it could be easily misunderstood that just by solving the obvious problems, the difficulties of existing as someone with DD in this society would also be eased. I feel that if more in-depth questions had been asked, that could have become a guide for tackling other related issues. (C2)
		Emotional barrier whilst cooperating (Q2-C-T3)	They asked me to use 'Discord' and I was troubled. I feel some resistance to something I have not touched before. There are people from different generations involved, so please make it accessible. (C1)
Changes in attitudes and behavior	Middle/High School students	Changing perceptions of developmental disability(Q3-H-T1)	At first I didn't feel familiar with it (DD) and had no idea about it, but it's not strange at all; and in a way, we all have DD. I don't think it's surprising to find them (PWDD) around me. (H8)
		Behavioral Changes (Q3-H-T2)	I've become sensitive to the term "DD". Whenever I see it on the news or on YouTube, I can't help but watch it. (H4)
		Changes in relationships (Q3-H-T3)	I used to think that they (PWDD) were scary or strange. After learning about DD through the program, I do not even feel scared. (H4)
		Future prospects (Q3-H-T4)	I now want to make society easier for people with DD and minorities to live in. (H4)
	University Students	Changing perceptions of developmental disability(Q3-U-T1)	At the time, the idea of social model was new. I have come to believe that I am responsible for how I can change the environment, because I too can exist as a element of the environment. (U1)
		Behavioral Changes (Q3-U-T2)	Now, when I happen to meet people in the gray zone, I am able to understand that they might have particular traits, and treat them with consideration. (U6)
		Changes in relationships (Q3-U-T3)	I am able to apply the lessons learned in GIP to my interpersonal relationships. I now have a different way of viewing relationships. I feel like this was an opportunity to become a more mature thinker. (U3)
	Cooperators	Increased awareness of contribution to their own activities (Q3-C-T1)	I felt sorry for the researchers (of DD) that there are not enough mutual support groups around the country for them too. I feel a sense of duty for what I am doing now. (C2)
		Reflection on how to deal with one's children (Q3-C-T2)	Personally I think, "I have to face my children". Thanks to the interview, there are instances where I feel I should not say too much (to my children). (C5)
		Hope for the future (Q3-C-T3)	A generation that has gone through the inclusive education curriculum is growing up, and I eagerly anticipate seeing the future society that these children will build. (C6)

Q1. Positive aspects

We asked interviewees ‘Tell us what you liked about i-GIP’ (Q1) and extracted 45 codes (Supplementary Material 2 (Code Table)). For middle/high school students, three themes were extracted. The themes were “Characteristics of Program” (Q1-H-T1), “Understanding of developmental disorders” (Q1-H-T2), and “Personal growth” (Q1-H-T3). Example sentences include "I was glad I got to listen to many lectures not only on DD but also on how to make presentations and prototypes and how to realize them (H5-Characteristics of program)".

Three themes “Use of project-based learning” (Q1-C-T1), “Raising awareness and understanding in different people” (Q1-C-T2), and “Attitude of students” (Q1-C-T3) were extracted from cooperators. One cooperator said “What I found most wonderful was that the students did not just listen passively but were proactive and engaged (in communicating with PWDD)” (C1-Use of project-based learning).

As can be seen, in Q1, responses from middle/high school students and university students regarding the program content and personal growth were similar. There were differences present too. For example, for the “Characteristics of Program” theme, middle/high schoolers focused on the opportunities i-GIP could offer outside of the traditional curriculum while university students found being able to work with middle/high school students a point of interest. For cooperators, we could conclude that they appreciated not only the content of the program itself but also the students’ attitude towards learning.

Q2. Areas of improvement

We asked interviewees ‘Please tell us if there are any improvements that need to be made for i-GIP’ (Q2) and extracted 28 codes (Supplementary Material 3 (Code Table)). In both middle/high school students and university students, the following themes were identified: “Challenges related to developmental disorders” (Q2-H-T1 and Q2-U-T1), “Administration” (Q2-H-T2 and Q2-U-T2), and “Capacity of participants” (Q2-H-T3 and Q2-U-T3). A notable difference for the theme “Challenges related to developmental disorders” was that while middle/high school students found conducting interviews difficult due to PWDD’s characteristics, university students found the topic of DD hard to learn and teach given its diverse and spectral nature, which stems from their perspective through managing and mentoring middle/ high school students. One university student shared “In terms of education for understanding disabilities, it is questionable whether it was good to rank the participants' proposed solutions. It is also true that competition is required for motivation. They were ranked according to whether it was feasible as a business, but the rankings do not reflect the severity of the problem” (U2-Administration).

For cooperators, apart from the theme, “Administration” (Q2-C-T1), which can be seen in middle/high school students, two other themes “Improvements solution-wise “(Q2-C-T2) and “Emotional barrier whilst cooperating” (Q2-C-T3) were extracted. Thus, from Q2, we identified areas for improvement not only with regard to program management, but also with regard to the ideas generated by the middle/high school students and the difficulty of addressing DD as a topic in an educational program.

Q3. Changes in attitudes and behavior toward DD

Middle/high school students and university students were asked two of the following questions: ‘Was there any change in your understanding of developmental disability before and after your participation in the program? If so, what kind?’ and ‘It has been a year since your participation in the program; has there been any change in your awareness of developmental disorders and behavior compared to before your participation? If so, what changes?’. As no differences were found in the answers to these questions (Q3), the answers were merged for analysis. Forty codes were extracted (Supplementary Material 4 ((Code Table)).

For middle/high school students and university students, three themes “Changing perceptions of developmental disorders” (Q3-H-T1), “Behavioral changes” (Q3-H-T2), and “Changes in relationships” (Q3-H-T3) were extracted. Apart from this, we extracted another code from middle/high school students: “Future Prospects” (Q3-H-T4). To provide a more detailed examination of these codes, we cite the perspectives of two participants. The first stated, “At first I didn't feel familiar with it (DD) and had no idea about it, but it's not strange at all; and in a way, we all have DD. I don't think it's surprising to find them (PWDD) around me.” (H8-Changing perceptions of developmental disability) and another, “I used to think that they (PWDD) were scary or strange. After learning about DD through the program, I do not even feel scared” (H4-Changes in relationships).

In Q3, middle/high school students reported not only changes in awareness and behavior toward DD, which was the topic of i-GIP, but also that what they had learned in i-GIP could be applied in their daily lives. In addition, some middle/high school students said that their experiences in i-GIP had influenced their career decisions.

We asked the cooperators: ‘It has been a year since you were involved in the program. Have you noticed any changes in yourself and the people around you in terms of attitudes and behaviors towards developmental disorders compared to before you were involved in the programme? If so, what changes?’ (Q3). Six codes and three themes “Increased awareness of contribution to their own activities” (Q3-C-T1), “Reflection on how to deal with one's children” (Q3-C-T2), “Hope for the future” (Q3-C-T3) were extracted (Supplementary Material 4 (Code Table)). One cooperator expressed, “A generation that has gone through the inclusive education curriculum is growing up, and I eagerly anticipate seeing the future society that these children will build” (C6-Hope for the future).

As can be seen in Q3, it is worth noting that there was a change in daily life for cooperators in terms of understanding and awareness of DD activities and parenting, as well as a newfound increased hope for the future of society.

## Discussion

The purpose of this study was to evaluate the validity and effectiveness of i-GIP. Through this study, we were able to extract the reasons for participants' participation, positive and negative aspects of i-GIP, and changes in participants' behaviors toward DD. These three objectives are discussed in turn below.

The good points of i-GIP reported by middle/high school students and university students were the “Characteristics of the program” (Q1-H-T1), “Understanding of DD” (Q1-H-T2), and their “Personal growth” (Q1-H-T3). This suggests that the characteristics of i-GIP met the expectations of the participants. i-GIP is a problem-solving program that allows participants to experience various activities, from interviews with PWDD and experts to presentations, and it can be said that these activities managed to match their reasons for participation. It has been reported that in Japan, the most common educational activities for understanding disabilities are simulation activities or joint learning in elementary and middle schools [17,18]. Furthermore, there are still challenges in developing programs that promote understanding of disabilities specific to DD [19]. As this program provides an experience outside of the regular school curriculum, we feel that it is not surprising that many participants felt that they had experienced personal growth and that their understanding of DD had been deepened through this program.

One of the good points reported by the cooperators was that through the program, young people were becoming involved in DD-related issues. Research has shown that the process of learning to understand disabilities should be supported by systematic and scientific information appropriate for children's stages of development [20,21]. However, there are few initiatives for the general public or in general education, in Japan [22], and the fact that this program targets middle/high school students itself was rare and thus a good point for the cooperators.

In addition to this, prior to their participation in i-GIP, some cooperators “thought that non-participants were “‘uninterested’ in developmental disorders”. This may be because they had witnessed or experienced behaviors that showed attitudes of “non-interference and indifference” by the general public [23]. On the other hand, some middle/high school students felt that PWDD “might get hurt because of getting involved with us”. This assumption of PWDD toward the general public and the resistance of the general public (the participating students in this case) with regard to being involved with PWDDs creates a misunderstanding within society and may be one of the factors reducing the interaction between the two groups. The increase in the general public’s interest in DD means that social misunderstanding and rejection, which are the sources of prejudice and discrimination [24], could possibly be gradually eliminated. 

Three points for improvement were raised by the middle/high school students. The first point was regarding the administration (Q2-H-T1), including “the downside of the business contest format”. Second, challenges related to DD (Q2-H-T2) were reported, including the fact that DD is “difficult to conceptualize” and that there were “limitations in the recruitment of interviewees”. Third, the limitations of the middle/high school participants’ capacity (Q2-H-T3) were reported, mainly the difficulty of “balancing the program and entrance exams”. Three points were also reported by the cooperators. First, they reported a need for change in the administration (Q2-C-T1), where “support for social implementation” and “avenues to disseminate information” were lacking. Secondly, the cooperators reported a desire for improvements in the solutions generated, (Q2-C-T2) citing a “lack of depth of understanding of issues”. Finally, for example, “difficulty in adapting to digital tools” was reported to contribute to the emotional barrier whilst cooperating (Q2-C-T3). Since many of these points are very pertinent, we believe that they should be paid attention to when designing future programs.

This study revealed a variety of changes in the participants' attitudes and behaviors toward DD. First, many reported that their knowledge had increased. According to Torii and Someki [25], those who have correct knowledge about ASD have less stigma; thus, the acquisition of correct knowledge is important for reducing prejudice. Given that currently half of the population in Japan has never had contact with people with disabilities [26] and that many participants had little prior knowledge of DD unless they had been personally acquainted with someone with DD, we can infer that the impact of this program was significant.

In addition, being able to “Put yourself into the shoes of someone with DD” was reported by both middle/high school students and university students. One contributing factor might be the emphasis on interviewing PWDD within the program. Previous research [27,28] has shown that interviews with PWDD are effective in reducing stigma. Because DD varies greatly from person to person, listening to the stories of individual participants, rather than learning only generalized knowledge and stories, may have led to a deeper understanding of the disabilities.

**Figure 2:**
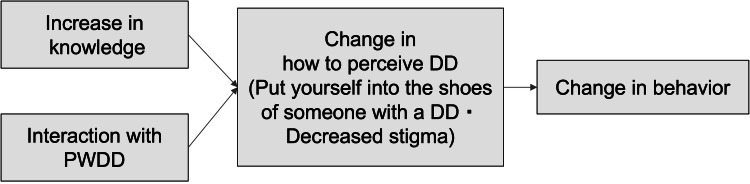
Changes of middle/high school participants and university students DD: Developmental Disorders, PWDD: Persons with Developmental Disorders

In addition to the change in awareness, the program also contributed to concrete changes in the behavior of the middle/high school students, as can be seen through the results: “I am now able to help students (PWDD) around me if they are in trouble” and “Learned to take into account the traits of others”. Thus, the changes in the middle/high school participants and the university students who managed the program can be summarized in Figure 2.

One of the sub-themes “I began to consider the circumstances of people who act in ways incomprehensible to me (e.g. whether they have unique characteristics)” is an example that shows that the middle/high school students and university students who interacted with PWDD through i-GIP were able to become more open-minded not only towards PWDD but also other people in general. This suggests that learning about disabilities and seeing the world through the perspective of people with disabilities through interviews will ultimately lead to the creation of a society that is welcoming of diversity.

Limitations

During the recruitment stage, i-GIP mainly focuses on recruiting students from elite schools. In addition, participants have to go through interviews before being selected for the program. Therefore, participants are likely to be highly motivated, and also able to have a good understanding of the educational program, which may lead to a positive bias. Therefore, if the average middle/high school student were to participate in i-GIP, the program may not be as effective as the reported results.

With regard to the interviews conducted by participants during i-GIP 2020, the PWDD who were able to be interviewed were limited to those who could reflect on and verbalize their experiences. As such, the information from PWDD was limited, and the participants might have only gained a partial understanding of DD. It is necessary to consider a system that would enable interviews about the problems of PWDD who are on the severe end of the spectrum or those who have difficulties regarding self-identity and verbal communication [29].

In addition, this study was conducted one year after the program ended. Therefore, it is possible that the results may be subject to recall bias.

## Conclusions

The purpose of this study was to review i-GIP, a problem-solving program targeting middle/high school students, and to clarify how i-GIP has changed the way in which participants view DD. While this study pointed out the positive effects of i-GIP, such as the participants learning to put themselves in the shoes of PWDD, it also reported some difficulties specific to dealing with DD. The strength of this study lies in its ability to conduct a comprehensive evaluation of i-GIP by interviewing three different groups: participating middle/high school students, university students involved in administration, and PWDD and their families (cooperators). We propose that regarding the topic of DD, a problem-solving program like i-GIP is more effective than classroom learning in terms of knowledge acquisition and the reduction of stigma. However, further research is required to verify its effectiveness. In order to provide this program to a wider audience, it is necessary to examine the method of implementation, duration, and target audience and consider packaging the program or introducing it into the school curricula.

## Appendices

**Table 4:** Attribute (Supplementary Material 1) PWDD: Persons with Developmental Disorders, H: Middle/High School Students, U: University Students, C: Cooperators

Participant No.	Three Groups	Age	Sex
H1	Middle/ High School Student	18	Female
H2	Middle/ High School Student	18	Female
H3	Middle/ High School Student	18	Male
H4	Middle/ High School Student	18	Female
H5	Middle/ High School Student	18	Female
H6	Middle/ High School Student	16	Female
H7	Middle/ High School Student	17	Female
H8	Middle/ High School Student	17	Female
U1	University Student	22	Male
U2	University Student	22	Male
U3	University Student	22	Male
U4	University Student	22	Male
U5	University Student	24	Male
U6	University Student	22	Male
U7	University Student	21	Female
C1	Cooperator (Parent of PWDD)	43	Female
C2	Cooperator (PWDD)	43	Male
C3	Cooperator (PWDD)	49	Female
C4	Cooperator (Parent of PWDD)	50	Female
C5	Cooperator (Parent of PWDD)	46	Female
C6	Cooperator (Parent of PWDD)	51	Female

**Table 5:** Positive Aspects (Supplementary Material 2) DD: Developmental Disorders, PWDD: Persons with Developmental Disorders

Three Groups	Theme	Sub-theme	Code
Middle/High School students	Characteristics of program	Organizing team was trustworthy	Organizing team was trustworthy
		Advantages of being held online	Could receive consultation online frequently
		First-hand experience of the problem-solving cycle	Could learn problem-solving techniques
			Learned how to look at things from multiple perspectives
			Could learn through lectures
		Comprehensive support	Ensured growth in participants
			Clear avenues for consultation
			Could work as a group with university students
	Understanding of developmental disorders	Learn about the actual lives/experiences of PWDD	I was able to hear from PWDD
			I was able to hear from stakeholders
			Learned the importance of thinking from the perspective of PWDD
	Personal growth	Broaden one's perspective	Exposure to various perspectives/viewpoints
		Fostered independence	Was able to think and act independently
University Students	Characteristics of program	Approach middle and high school students	Participants are middle and high school students
		Long-term project	Long-term involvement in the project led to changed attitudes towards DD in middle/high school students
		Advantages of being held online	Was held online
		First-hand experience of the problem-solving cycle	The problem-solving approach allowed for in-depth collaboration with PWDD
			The program followed a cycle of problem identification, solution devising, and implementation
		Prior training provided sufficient preparation for the program	Mentors-only trial run was helpful in the implementation of the actual program
		Managed to accomplish something I would not have been able to do alone	I learned more about DD than I could have learned on my own
	Understanding of developmental disorders	Learn about the actual lives/experiences of PWDD	Was able to work with people with DD
			Interviews gave me an insight into the realities of DD
		Better understanding of DD	I was able to put myself in the shoes of the parties involved by hearing their stories directly
			Was able to involve a variety of people
			I learned the importance of working with PWDD
			University students also gained a better understanding of DD
	Personal growth	Broaden one's perspective	More places to draw ideas from
		Become more accepting of diversity	Become more interested in other disabilities
			Become more accepting of diversity
		Influenced my image of my future	Gained an idea of what I want to pursue in the future
Cooperator	Use of project-based learning	Learn collaborative skills	I was thinking about how to solve the problem and with whom to cooperate
		Participants can consider issues from the perspective of PWDD	Participants can evaluate issues related to PWDD
		Solutions were highly practical and practicable	Quality of ideas were good
			They used methods used by companies
			They worked with companies to increase the feasibility of the project
	Raising awareness and understanding in different people	Effect of program being held online	The online format allowed for remote participation
			I was able to influence people who were not directly involved
		Significance of collaborating with people not directly connected to DD	I find it meaningful that even people not directly connected to DD can become involved
		Young people are working on DD	I was happy DD was chosen as the topic
			Students showed interest (in DD)
			It's reassuring to hear that students are involved (with DD)
	Attitude of students	I learned of potential supporters	I learned that there are people who really care about DD
		Participants listened attentively	Students kept an open mind regarding DD
		I learned something from the students	I learned that there were students who were willing to tackle challenges
			I learned from the students’ event management skills and positive attitudes

**Table 6:** Areas of Improvement (Supplementary Material 3) DD: Developmental Disorders, PWDD: Persons with Developmental Disorders

Three Groups	Theme	Sub-theme	Code
Middle/ High school students	Challenges related to developmental disorders	Difficulties in interviewing PWDD	Characteristics of PWDD led to some challenges in the interview process
	Administration	Difficulties of holding program online	It was much more lecture-based because it wad online
			Wanted to work on it in person
			Wanted to interact more with other teams
		The downside of the business contest format	Wanted to know about the process of the project rather than just the final product/presentation of other teams
		Ways to contact the management	There was not always a prompt response from the administration
	Capacity of participants	There were too many tasks to complete	It was difficult to grasp the big picture because we were caught up in the task at hand
		Balancing the program and entrance exams	Had to quit the project to take the entrance exam
University students	Challenges related to developmental disorders	Difficult to conceptualize DD	DD being on a spectrum made it difficult to create a systemic/cohesive representation of DD
		Overabundance of information	It was difficult to estimate the amount of prior knowledge needed regarding DD
		Wide range of traits	Because of the wide range of characteristics of DD, it was difficult to settle on one general explanation
		Limitations in the recruitment of interviewees	Only a limited number of PWDD could be interviewed
		Difficulties in communication	There was anxiety and unease about the difficulty of communication during the interviews
		Insufficient consideration to PWDD	Insufficient consideration to PWDD
	Administration	Balance between quantity and quality of interviews	More interviews did not mean better
		The downside of the business contest format	It is questionable whether it was appropriate to rank the proposed solutions to the issues
			There was not much interaction among the teams due to the business contest format
		Difficulties of holding program online	Because the event was held online, it was difficult for the teams to interact with each other
			Because it was held online, it was difficult to have a more in-depth discussion
			Management of remote participants was difficult
		Limitation on number of participants	The number of participants was limited
	Capacity of participants	Work-life balance	I had to cut back on sleep to complete the tasks
Cooperators	Administration	Lack of support for social implementation	There was insufficient support for ideas post-program
		Lack of avenues to disseminate information	Insufficient ability to provide information to those who needed it
		Burden of university students in management	I thought college students had a hard time
	Improvements solution-wise	Lack of depth of understanding of issues	Failure to address deeply hidden underlying issues
	Emotional barrier whilst cooperating	Difficulty in adapting to digital tools	I was confused about how to use digital tools
		Worries about whether they are cooperating well	I was worried about whether I was cooperating well

**Table 7:** Changes in Attitudes and Behavior Toward Developmental Disorders (Supplementary Material 4) DD: Developmental Disorders, PWDD: Persons with Developmental Disorders

Three Groups	Theme	Sub-theme	Code
Middle/High School students	Changes in perceptions of DD	Increased knowledge of DD	Increased knowledge of DD
			Increased knowledge of disabilities in general
		Became aware of DD in daily life	I felt closer to PWDD
			Increased awareness of DD in daily life
		Put yourself into the shoes of someone with DD	Learned to put myself in PWDD's shoes
		Changed my awareness about DD in my daily life	Became more negative about the voices around me
		I realized I didn't understand DD	Realised the difference between what I had imagined and what issues PWDD face in actuality
		I can now see DD as an individual characteristic	I can now see DD as a trait
	Behavioral Changes	I am now able to respond to DD appropriately	Able to help students around me when they are in trouble
		I'm able to have in-depth interactions with PWDD	I learned about individuals and was able to talk to people with disabilities without fear of hurting them.
		I've learned to use language that takes PWDD into account	I've learned to use language that takes PWDD into account
		Encouraged others to use language that takes PWDD into account	Based on the knowledge gained from the program, I corrected the way the school teacher talked
		Worked to contribute to DD in society	Worked on a project to reduce the probability of traffic accidents for the parties involved
			I wrote a feature on disability for the school newspaper
		Became interested in ways to make a positive social contribution	I'm willing to support them should the opportunity arise
		Became actively exposed to information on DD	When I see news on DD online, I read them
			I began to research inclusive education
	Changes in relationships	I began to consider the circumstances of people who act in ways incomprehensible to me (e.g. whether they have unique characteristics)	I began to think about the context of (other's) actions
			I no longer feel fear when I see unusual people in my daily life
	Future prospects	Wish to create a livable society for all	I wanted to create a society where minorities can live easily
		Wish to create a livable society for all	I've learned that I want to live comfortably
		Decided on my future path	I have decided on the university and department of my choice
University Students	Changes in perceptions of DD	Increased knowledge of DD	Increased knowledge of DD
		See DD as a part of diversity	See DD not as a disability but also as a part of diversity
		See DD as an individual characteristic	
		No feelings of resistance towards DD	I no longer feel a strange resistance when I hear DD
		Can put oneself in shoes of PWDD	I learned about the social model of disability and was able to see DD as relevant to my own life
		Developed interest in DD	The world of PWDD became an object of my intellectual curiosity
		Changed my awareness about DD in my daily life.	Increased awareness of DD in daily life
		Sustained change in mindset toward DD	Persistence of attitudes toward developmental disorders that changed before and after the program
			You may forget the details, but the fundamental ideas remain
	Behavioral Changes	I've learned to help people who seem to have characteristics (of DD) if they are in need	I've learned to help people who seem to have developmental disorders if they are in trouble
		Learned to take into account the traits of others	Learned to take into account the traits of others
		I started to think about what I could do for people who might be on the spectrum	I started to think about what I could do for people who are on the spectrum
		More opportunities to work with PWDD	I come into more contact with people with DD
		Became actively exposed to information on DD	When I see news on developmental disorders in the online, I read about them
		I think less and less about DD as time goes on	I think less about developmental disorders than during the program
	Changes in relationships	Realization that everyone is fine just as they are	Take things as my own business, and that all people are fine just as they are.
		I began to consider the circumstances of people who act in ways incomprehensible to me (e.g. whether they have unique characteristics)	Tolerance for people with or without DD who behave in ways I can't imagine
		Increased interest in disabilities from social model perspective	Interest in not only DD, but also in disabilities that are largely influenced by the environment
		Better interpersonal relationships.	The ideas I learned at the program are being applied in my daily interpersonal relationships
		Started to think too much about the other person's background in interpersonal relationships	I can't get mad at people anymore because I think too much about how much I should consider when talking to them
Cooperators	Increased awareness of contribution to their own activities	Take on new challenges in their own activities	I was hesitant to reach out to people not involved with DD, but now I have the courage to take the first step
		Sense of purpose in their activities	I wanted to plan more opportunities for people to meet PWDD
	Reflection on how to deal with one's children	I reconsidered how to deal with my own children	Gave me time to rethink my own child's DD
			Provided me an opportunity to reflect on how I deal with my own children
	Hope for the future	Hope for future society	Expectations for inclusive education, etc. have been raised
		Budding hope	Open-mindedness of the students in this program has led to parents of kids with DD feeling hopeful for the future
